# Incidence of and risk factors for vaginal cuff dehiscence following total laparoscopic hysterectomy: a monocentric hospital analysis

**DOI:** 10.1007/s00404-021-06064-0

**Published:** 2021-05-03

**Authors:** Julia Caroline Radosa, Marc Philipp Radosa, Julia Sarah Maria Zimmermann, Eva-Marie Braun, Sebastian Findeklee, Annette Wieczorek, Lisa Stotz, Amr Hamza, Ferenc Zoltan Takacs, Uda Mareke Risius, Christoph Gerlinger, Christoph Georg Radosa, Stefan Wagenpfeil, Erich-Franz Solomayer

**Affiliations:** 1 Department of Gynecology and Obstetrics, Saarland University Hospital, Kirrbergerstraße 100, 66421 Homburg, Saar Germany; 2 Department of Gynecology and Obstetrics, Klinikum Bremen–Nord, Bremen, Germany; 3 Department of Business and Psychology, University of Applied Sciences Europe, Berlin, Germany; 4 Department of Radiology, Dresden University Hospital, Dresden, Germany; 5 Institute of Medical Biometry, Epidemiology and Medical Informatics, Saarland University Hospital, Homburg, Saar Germany

**Keywords:** Vaginal cuff dehiscence, Total laparoscopic hysterectomy, Laparoscopic surgery, Risk factors, Complication, Gynecologic surgery

## Abstract

**Purpose:**

Vaginal cuff dehiscence (VCD) is one of the major surgical complications following hysterectomy with data on incidence rates varying largely and studies assessing risk factors being sparse with contradictive results. The aim of this study was to assess the incidence rate of and risk factors for VCD in a homogenous cohort of women treated for benign uterine pathologies via total laparoscopic hysterectomy (TLH) with standardized follow-up.

**Methods:**

All patients undergoing TLH at the Department of Gynecology and Obstetrics, Saarland University Hospital between November 2010 and February 2019 were retrospectively identified from a prospectively maintained service database.

**Results:**

VCD occurred in 18 (2.9%) of 617 patients included. In univariate and multivariate analyses, a lower level of surgeon laparoscopic expertise (odds ratio 3.19, 95% confidence interval (CI) 1.0–9.38; *p* = 0.03) and lower weight of removed uterus (odds ratio 0.99, 95% CI 0.98–0.99; *p* = 0.02) were associated positively with the risk of VCD.

**Conclusion:**

In this homogenous cohort undergoing TLH, laparoscopic expertise and uterine weight influenced the risk of postoperative VCD. These findings might help to further reduce the rate of this complication.

## Introduction

Hysterectomy ranks among the most commonly performed gynecological surgical procedures [1]. Nearly 150,000 hysterectomies are performed annually in Germany [2].

Eighty-five percent of these surgeries are elective interventions, performed due to benign uterine pathologies [3]. Vaginal hysterectomy has been the traditional standard-of-care procedure for surgical uterus removal, but a gradual shift has occurred in surgical gynecology in the past 2 decades from vaginal and abdominal approaches to minimally invasive techniques, with increased performance of total laparoscopic hysterectomy (TLH) [4]. Major advantages of TLH over open procedures are reduced postoperative pain, faster recovery, and shorter hospitalization [5]. However, a notable disadvantage is the higher incidence of vaginal cuff dehiscence (VCD), defined as the separation of the previously sutured vaginal incision [6]. Although VCD is a rare complication, the evisceration of abdominal or pelvic organs through the vaginal breach can lead to serious sequelae, such as bowel injury, peritonitis, necrosis, and sepsis; regardless of its extent, VCD necessitates additional surgery in most cases [7, 8]. VCD occurs significantly more frequently after TLH (0.64–5.4%) than after abdominal (0.38%), vaginal (0.11%), and laparoscopically assisted (0.46–1.5%) procedures [9, 10]. Electrosurgery, previous radiation therapy, pelvic floor defects, and early postoperative sexual intercourse have been associated with the occurrence of VCD in general, but data on surgery-related risk factors for post-TLH VCD are sparse and contradictory [2, 11, 12]. Given the paucity of data and the high variation on the incidence of VCD following TLH in the current literature, we aimed to assess the frequency of and risk factors for VCD following TLH performed for benign uterine pathologies in a large cohort treated at a laparoscopic tertiary referral center.

## Materials and methods

All patients who underwent TLH due to benign uterine disorders between November 2010 and February 2019 at the Department of Gynecology and Obstetrics, Saarland University Hospital, Homburg, Germany, were identified retrospectively through a prospectively maintained service database. The study was approved by the Saarland Institutional Review Board (Reference No. 85/16) and registered with the German Clinical Trials Register (DRKS) (No. DRKS00009904).

All methods were carried out in accordance with respective guidelines and regulations. All TLHs were performed under general anesthesia at Department of Gynecology and Obstetrics at Saarland University Hospital Homburg, Germany, where the procedure has been the standard surgical approach for hysterectomy since 2009. The surgical techniques applied are described in detail elsewhere [13–15]. Preoperatively, all patients provided medical histories and underwent gynecological examination, transvaginal ultrasound, and ultrasound of the kidneys. Basic laboratory tests were performed on admission.

All procedures were performed using a vaginal manipulator (Hohl; Karl Storz SE & Co. KG, Tuttlingen, Germany). Colpotomy of the vaginal fornix was performed with a monopolar hook (Karl Storz SE & Co. KG, Tuttlingen, Germany). The vaginal vault was closed with single-layer laparoscopic suturing (single knot or running) using Vicryl 1-CT-1 (Ethicon Inc., Somerville, NJ, USA) and an intracorporeal knot technique. All patients received perioperative antibiotics (cefuroxime, 1.5 g; Fresenius Kabi, Bad Homburg, Germany), indwelling urinary catheters, and low-molecular-weight heparin (enoxaparin sodium, 40 mg; Sanofi, Paris, France) as thromboembolism prophylaxis postoperatively. All patients underwent standardized follow-up, including gynecological examination and transvaginal sonography, at 6 weeks postoperatively, and were interviewed by telephone about the occurrence of VCD. Patients with incomplete datasets, including missing follow-up information, were excluded from the study.

VCD was defined as full-thickness separation of the anterior and posterior edges of the vaginal cuff, with or without bowel evisceration categorized as complete when the separation involved the entire length of the vaginal vault and as partial when it involved only part of the incision [11, 16]. Evisceration was defined as the expulsion of abdominal content through a vaginal cuff defect. VCD repair was conducted laparoscopically with complete inspection of the abdominal cavity for hematoma, abscess, or bowel injury, followed by irrigation, excision of necrotic tissue, and re-suturing using single-knot technique with Vicryl 1-CT-1 (Ethicon Inc., Somerville, USA). In cases of minimal dehiscence and no clinical or sonographic suspicion of hematoma, abscess, or bowel injury, vaginal repair with single-knot suturing was performed.

Clinical data comprising patients’ age (years), body mass index (kg/m^2^), menopausal status, childbirth (number, mode of delivery) and surgeries performed in the past (measured by a surgery score: 0 points: no previous surgery, 1 point: previous laparoscopic surgery 2 points: open surgery, e.g., laparotomy, cesarean section) described by Boosz et al. and surgical parameters such as duration of surgery (minutes), performance of consecutive adhesiolysis or ureterolysis, suturing technique (single stitches vs. running non-overcast suture), duration of surgery (minutes), postoperative complications (according to the Clavien–Dindo classification of surgical complications), interval between surgery and first sexual intercourse (weeks), time between surgery and detection of VCD (days) and laparoscopic expertise of the surgeon assessed according to the GESEA program (Gynaecologic Endoscopic Surgical Education and Assessment) of the European Academy of Gynaecological Surgery and the European Society for Gynaecologic Endoscopy (ESGE) (GESEA level 1 (Bachelor) and GESEA level 2 [minimal invasive Gynaecological Surgeon (MIGS)] [17–20].

### Statistical analysis

Data were collected in an Excel database (Excel 2014, Microsoft Corporation, Redmond, WA, USA). The Kolmogorov–Smirnov test was used to assess normality distribution for quantitative variables. As the data were non-normally distributed, we used the Mann–Whitney *U* test to assess differences between groups. For categorical variables, we used Pearson’s Chi-squared test for group comparisons. For multiple analysis, binary logistic regression with stepwise forward and backward selection was used to identify factors and possible confounders associated with the occurrence of VCD. Statistical tests were two-sided and subject to a significance level of 5%. Due to the explorative nature of the investigation, we did not account for the issue of multiple testing and thus report unadjusted *P* values. The statistical analyses were performed using SPSS (Version 19; SPSS Inc., Chicago, IL, USA).

## Results

### Patient characteristics

Six hundred and sixty-three patients who received TLH for benign uterine pathologies between November 2010 and September 2018 at the Department of Gynecology and Obstetrics, Saarland University Hospital, Homburg, Germany were identified from the service database. Four patients were excluded because of malignant final histology reports, 18 patients were excluded because of non-attendance of 6 weeks postoperative follow-up visits, and 24 patients were excluded because of missing further postoperative information on occurrence of VCD, leaving a total of 617 patients included in the final analysis. Detailed patient characteristics and surgical parameters are shown in Tables 1 and 2. Postoperative VCD occurred in 18 patients (incidence rate, 2.9%). Median time between surgery and detection of VCD were 12 days (range 0–69). Patients with VCD presented with vaginal bleeding or discharge (*n* = 12), abdominal pain (*n* = 5), and vaginal pressure (*n* = 1). Three patients had complete VCD, with the evisceration of abdominal content through the dehiscence in two cases. Sixteen patients with VCD required re-operation, via laparoscopy in 13 patients and via single-knot vaginal closure in 3 patients with minor dehiscence (Table 3).

**Table 1:** Patient’s characteristics (*n* = 617)

	*n* = 617
	Median (Min–Max)
Age (years)	47 (26–82)
BMI (kg/m^2^)	25.7 (19–54.6)
Parity	1 (0–7)
Number of vaginal deliveries	1 (0–7)
Previous surgery score	1 (0–16)
	*N* (%)
Menopausal status	
Pre-/perimenopausal	494 (80)
Postmenopausal	123 (20)
Smoker	109 (18)
Main indications for hysterectomy (%)	
Symptomatic uterine fibroids	366 (59)
Endometriosis	110 (18)
Cervical dysplasia	49 (8)
Uterine prolaps	48 (8)
Other	44 (7)

**Table 2:** Surgical outcomes (*n* = 617)

	Median (Min–Max)
Surgical parameters	
Duration of surgery (min)	109 (40–390)
Hemoglobin drop (g/dl)	1.1 (0–6.9)
Postoperative hospitalization (days)	3 (1–25)
Uterine weight (g)	169 (19–2148)
	*N* (%)
Adhesiolysis	274 (44)
Ureterolysis	287 (47)
Surgeons laparoscopic expertise	
GESEA level I	297 (48)
GESEA level II	320 (52)
Suturing technique	
Single knot suture	519 (84)
Running suture	98 (16)
Postoperative complications (Clavien–Dindo)	
Mild complications (I–II)	16 (2.6)
Severe complications (III–V)	20 (3.2)
First postoperative sexual intercourse < 6 weeks	119 (19)

**Table 3:** Incidence and presentation of VCD (*n* = 617)

Vaginal cuff dehiscence (VCD)	18 (2.9 %)
Interval between surgery and occurrence of VCD [days; median (range)]	12 (0–69)
	*n*=18
Symptom presented with for VCD	
Vaginal bleeding	10 (55 %)
Pain	7 (39 %)
Vaginal pressure	1 (6 %)
Type of dehiscence	
Partial dehiscence	15 (83 %)
Complete dehiscence	3 (17 %)
Evisceration	
Yes	2 (11 %)
No	16 (89 %)

### Risk factor analysis

In the univariate analysis, uterine weight and surgeon’s laparoscopic expertise were associated with the incidence of VCD. The median weight of removed uterus was significantly lesser among patients with than among those without VCD [91 g, (range 55–321) vs. 171 g, (range 19–2148), *P* ≤ 0.01]. Significantly more patients with than without VCD were operated on by level 1 surgeons [14 (78%) vs. 4 (22%), *P* ≤ 0.01] (Table 4). On multivariate analysis, the risk of VCD was associated with a lower level of surgeon laparoscopic expertise [odds ratio 3.19 (95% CI 1.0–9.38); *P* = 0.03] and lesser weight of removed uterus [odds ratio 0.99 (95% CI 0.98–0.99); *P* = 0.02]. The incidence of VCD was not associated with the duration of surgery, body mass index, or suturing technique (Table 5).

**Table 4:** Univariate analysis of patient’s characteristics and surgical outcome patients with vaginal cuff dehiscence (VCD) versus patients without VCD (*n* = 617)

	No VCD *n* = 599	VCD *n* = 18	*P*
	Median (min–max)		
Age (years)	46 (26–82)	45 (29–72)	0.75
BMI (kg/m^2^)	25.8 (19–54.6)	24.7 (20.6–48.2)	0.29
Parity	1 (0–7)	1.5 (0–3)	0.68
Number of vaginal deliveries	1 (0–7)	0.5 (0–2)	0.52
Previous surgery score	1 (0–16)	1 (0–14)	0.13
	*N* (%)		
Smoker	105 (18)	4 (22)	0.36
Menopausal status			0.49
Pre-/perimenopausal	480 (80)	14 (78)	
Postmenopausal	119 (20)	4 (22)	
Main indications for hysterectomy (%)			0.26
Symptomatic uterine fibroids	359 (60)	7 (39)	
Endometriosis	104 (17)	6 (33)	
Cervical dysplasia	47 (8)	2 (11)	
Uterine prolaps	46 (8)	2 (11)	
Other	43 (7)	1 (6)	
	Median (min–max)		
Surgical parameters			
Duration of surgery (min)	107 (40–390)	104 (47–281)	0.51
Hemoglobin drop (g/dl)	1.2 (0–6.9)	1.3 (0–3.7)	0.6
Postoperative hospitalization (days)	3 (1–25)	4 (2–14)	0.29
Uterine weigth (g)	171 (19–2148)	91 (55–321)	** ≤ 0.01**
	*N* (%)		
Adhesiolysis	268 (45)	6 (33)	0.34
Ureterolysis	278 (46)	9 (50)	0.76
Surgeons laparoscopic expertise			** ≤ 0.01**
GESEA Level I	283 (47)	14 (78)	
GESEA Level II	316 (53)	4 (22)	
Suturing technique			0.22
Single knot suture	502 (84)	17 (94)	
Running suture	97 (16)	1 (6)	
First postoperative sexual intercourse < 6 weeks	126 (21)	2 (11)	0.26

**Table 5:** Multivariate analysis of factors associated with the incidence of vaginal cuff dehiscence

	Odds ratio (95% CI)	*P*
Surgeons laparoscopic expertise (GESEA level I vs. level II)	3.19 (1.0–9.38)	0.03
Uterine weigth (g)	0.99 (0.98–0.99)	0.02
Suturing technique (single-knot suture vs. running suture)	2.83 (0.37–21.87)	0.32
BMI (kg/m^2^)	0.97 (9.0–1.05)	0.47
Duration of surgery (min)	0.99 (9.99–1.01)	0.59

## Discussion

In designing the present study, we sought to evaluate incidence and risk factors of VCD following total laparoscopic hysterectomy for benign uterine pathologies in a large cohort treated at a laparoscopic tertiary referral center, with a standardized follow-up. We found a rate of 2.9% for vaginal cuff dehiscence, which is in line with vaginal cuff dehiscence rates described in the literature of 0.64–5.4%. Low surgeon laparoscopic expertise and low uterine weight were identified as risk factors for VCD. Two recent studies investigated this subject using similar designs [8, 11].

Rettermaier et al. conducted a retrospective analysis including 1876 patients undergoing TLH or robotic-assisted laparoscopic hysterectomy at a single institution and found a VCD incidence rate of 0.75% (*n* = 14), which was lower than in this study (2.9%). These discrepancies may be, possibly related to differences in study design. Two-thirds of procedures in that study were robotic-assisted laparoscopic hysterectomies, for which lower VCD rates (0.4–4.1%) have been reported than for laparoscopic hysterectomy [21, 22]. In addition, the authors used barbed sutures for vaginal vault closure in most cases (no VCD occurred following this approach) and identified Vicryl suture use as a VCD risk factor [8]. Given the growing body of evidence confirming the protective effect of the usage of barbed suture to prevent VCD, this might be a further explanation for the low VCD rate observed by the authors [23–25]. Finally, Rettermaier et al. identified VCD cases using coding data, which might have introduced selection bias and led to VCD underreporting due to inadequate procedure coding and loss to follow-up.

In a retrospective multi-institutional analysis including 12,398 patients undergoing hysterectomy (laparoscopic, vaginal or abdominal), the VCD rate was significantly higher for TLH (0.64%) than for abdominal and vaginal hysterectomies (0.2% and 0.13%), respectively, and laparoscopic vaginal cuff closure during TLH (performed in 20/38 VCD cases) was the main risk factor for VCD [11]. The VCD rate for TLH with vaginal closure (0.24%) was similar to those for abdominal and vaginal hysterectomies. Differences in the VCD rate between that study and ours might be due to differences in assessment of VCD and follow-up. Surgeons’ laparoscopic expertise may also have contributed, although the authors did not provide such information.

Higher VCD rates following TLH with laparoscopic (vs. vaginal) vault closure have been reported in institutions with moderate laparoscopic expertise [26]. On the contrary, a prospective randomized trial conducted by the Italian Society of Gynecologic Endoscopy including only high-volume (> 500 gynecological interventions/year) Italian referral centers [27]. These results are in line with our finding that greater laparoscopic expertise showed a protective effect against incidence of VCD. Surgeon’s experience with a surgical technique has been shown to have major impact on surgical outcomes and complication rates [20, 28]. Particularly laparoscopic suturing requires advanced training, and low proficiency may compromise the quality of vaginal cuff closure. Thus, reported differences in the incidence of VCD according to surgical technique might reflect surgeons’ expertise with the respective surgical approach, underlining the importance of training programs for minimally invasive surgeries [29].

We identified an inverse association between uterine weight and the incidence of VCD, which to our knowledge has not been reported previously [11, 29]. Greater mean uterine weights (> 300 g) in VCD groups in previous studies assessing VCD rates discussed above, compared to the median weight of 120 g in the VCD group reported in our study, may have prevented identification of this variable as a risk factor. Our finding may be explained by the use of a small uterine-manipulator portio cap (32 mm) for patients with small uteri.

**Fig. 1:**
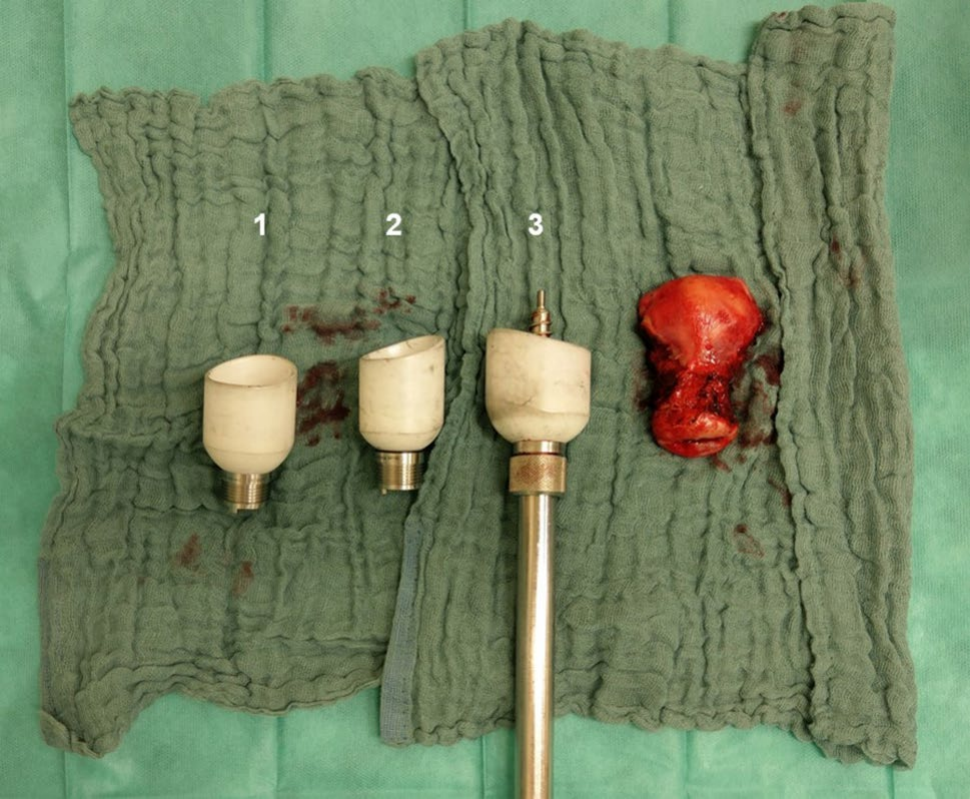
Different portio caps used for total laparoscopic hysterectomy ((1)–32 mm, (2)–35 mm, and (3)–40 mm)

The usage of the small cap might lead to two possible complications explaining the higher VCD rate in this group. First in some cases, the small cap does not fully enclose the whole cervix, leading to vaginal vault opening above the level of the vaginal fornix, leaving cervical tissue in the vaginal vault (Fig. 1). This tissue might not adapt well, leading to necrosis and a higher VCD rate. In addition, smaller vaginal-tissue resection in patients with smaller uterus harbors the danger of greater thermal damage. These observations should be kept in mind for women with smaller uterus, who are typically not regarded as being at increased risk of postoperative complications. Special attention should be given to uterine-manipulator cap selection in these patients and cap placement should be checked for full uterine portio enclosure after uterine-manipulator application.

This study has several limitations. It was retrospective and may have involved reporting bias. However, patient identification from a prospectively maintained service database, and the standardized follow-up, enhanced the study design and minimized the risk of VCD underreporting. This approach might also explain the higher VCD incidence than reported previously. Moreover, due to our standardized use of surgical suturing material, our findings might not be applicable to different suturing circumstances. Giving the possible protective effect of barbed suture use on VCD, this might impact the future incidence of VCD [8, 23, 24].

Since the median interval between surgery and occurrence of VCD were 12 days (0–69), most VCD were detected before the follow-up visit 6 weeks postoperatively and an earlier follow-up might have been more accurate. Another limitation of this study is the sample size. Although our sample was not small relative to those of other studies addressing similar questions, the rareness of VCD renders its statistical correlation with risk factors difficult [9, 24].

## Conclusion

We found a VCD rate of 2.9% in this homogenous cohort treated with TLH for benign uterine pathologies at a single institution with a standardized follow-up and identified low surgeon’s laparoscopic expertise and low uterine weight as factors associated with an increased risk of VCD. These findings emphasize the influence of surgeon’s experience on the occurrence of VCD and help to identify patients with small uterus as an at-risk population. Our observations provide insight to minimize the risk of VCD, but additional prospective research is needed.

## Data Availability

The dataset used and analyzed during the current study is available from the corresponding author on reasonable request.
